# Current News

**Published:** 1892-11

**Authors:** 


### Current News.

### COMMITTEE ON ESSAYS (No. 17) OF THE WORLD'S COLUMBIAN DENTAL CONGRESS.

### PRELIMINARY NOTICE.

It is the aim of the World's Columbian Exposition "to gather together evidences of the material progress, achievement, and civilization of the world, and so arrange them that every department of human endeavor may be studied and examined through all its various grades of development." In recognition of the important part which the growth and professional development of dental science and art have contributed to the world's progress, the holding of a "World's Columbian Dental Congress" under the auspices of the World's Congress Auxiliary has been provided for by a duly authorized general executive committee, appointed by the dental profession of America through its two governing dental organizations. The undersigned committee, being intrusted through authority of the general executive committee with the duty of soliciting essays appropriate to the "Division of Dental and Oral Surgery," and realizing the necessity and great importance of a proper presentment of the literary and scientific phases of our profession through the contributions of its representative men, respectfully invite the preparation of papers directly or collaterally connected with the above-named department, to be submitted to this committee at least three months previous to the convening of the congress, the date of which will be August 17, 1893. All essays must be in the hands of the committee at the time requested, in order that opportunity shall be afforded for their arrangement and assignment to appropriate sections. Those contributions earliest received will, of course, take precedence. The committee especially desires that answers to this request, including the titles of papers, shall be forwarded to the chairman at as early a date as possible. The authors of those papers which are accepted by the committee for a place upon the programme will be notified of the fact as promptly as possible. Information respecting the special prize essay on oral hygiene may be obtained from the chairman of the Committee on Prize Essay (No. 23), Dr. Theodore Stanley, Kansas City, Mo.; information respecting essays in general, from the chairman of the Committee on Essays (No. 17). Forward all communications to the chairman of the Committee on Essays, Lock Box 1615, Philadelphia, Pa.

Dr. Edward C. Kirk, *Chairman*, Philadelphia, Pa.
Dr. J. W. Wassall, Chicago, Ill.
Dr. A. H. Thompson, Topeka, Kan.
Dr. H. H. Johnson, Atlanta, Ga.
Dr. L. G. Noel, Nashville, Tenn.
Dr. C. Newlin Pierce, Philadelphia, Pa.

---

### RECENT PATENTS.

A list of recent patents, reported specially for the International Dental Journal.

477,791.—Dental Reflector and Tongue-Guard. Walter E. Andrews, New York, N. Y. Filed February 25, 1892.

478,217.—Dental Forceps. Charles E. Blake, Sr., San Francisco, Cal. Filed October 22, 1891.

478,312.—Clamp for Dental Flasks. Morgan R. B. Creery, Ebensburg, Pa. Filed December 5, 1891.

478,343.—Dental Tool. Charles P. Lennox, Toronto, Canada. Filed December 11, 1891.

478,589.—Dental Chair. William H. Gilbert, Philadelphia, Pa., assignor to the S. S. White Dental Manufacturing Company, same place. Filed March 24, 1890.

478,590.—Dental Chair. Aaron P. Gould, Canton, Ohio. Filed June 5, 1890.

478,672.—Dental Chair. Basil M. Wilkerson, Baltimore, Md., assignor, by mesne assignments, to the S. S. White Dental Manufacturing Company, Philadelphia, Pa. Filed December 21, 1877.

478,881.—Dental Tool. Edward C. Moore, Detroit, Mich. Filed January 29, 1892.

Designs.—21,666.—Dental Cabinet. Josiah O. Keller, Fort Wayne, Ind. Filed May 14, 1892. Term of patent, fourteen years.

478,992.—Dental Engine. Frank A. Damond, Fitchburg, Mass. Filed April 6, 1892.

481,164.—Dental Engine. F. A. Damond, Fitchburg, Mass. Filed July 16, 1891.

481,479.—Dental Clamp. J. S. Deardorff, Canal Dover, Ohio. Filed March 28, 1892.

Trade-Mark. 21,733. Dentifrice. Florence Manufacturing Company, Northampton, Mass., New York, N. Y., and Chicago, Ill. Filed June 20, 1892. Essential feature, the words "Twin Luxuries."

---

### BOARD OF EXAMINERS OF DISTRICT OF COLUMBIA.

Appointments made by the Commissioner of the District of Columbia to the first "Board of Dental Examiners:"

William Donnally, D.D.S., for five years; H. M. Schooley, D.D.S., for four years; J. Roland Walton, D.D.S., for three years; H. B. Noble, D.D.S., for two years; John B. Rich, D.D.S., for one year.

The board organized by electing J. Roland Walton, President; William Donnally, Secretary.

---

### WORLD'S COLUMBIAN DENTAL CONGRESS OFFICERS ELECTED AT CHICAGO.

*President*, L. D. Shepard, Boston, Mass. *Vice-Presidents*, W. W. H. Thackston, Farmville, Va.; A. L. Northrop, New York, N. Y.; W. H. Morgan, Nashville, Tenn.; W. W. Allport, Chicago, Ill.; W. O. Kulp, Davenport, Iowa; C. S. Stockton, Newark, N. J.; Edwin T. Darby, Philadelphia, Pa.; H. J. McKellops, St. Louis, Mo.; J. Taft, Cincinnati, Ohio; J. H. Hatch, San Francisco, Cal.; J. B. Patrick, Charleston, S. C.; J. C. Storey, Dallas, Texas. *Secretary-General*, A. W. Harlan, Chicago, Ill. *Assistant Secretaries*, Geo. J. Friedrichs, New Orleans, La.; Louis Ottofy, Chicago, Ill. *Treasurer*, J. S. Marshall, Chicago, Ill.

